# ATOR-1017 (evunzekibart), an Fc-gamma receptor conditional 4-1BB agonist designed for optimal safety and efficacy, activates exhausted T cells in combination with anti-PD-1

**DOI:** 10.1007/s00262-023-03548-7

**Published:** 2023-10-05

**Authors:** Karin Enell Smith, Sara Fritzell, Anneli Nilsson, Karin Barchan, Anna Rosén, Lena Schultz, Laura Varas, Anna Säll, Nadia Rose, Maria Håkansson, Laura von Schantz, Peter Ellmark

**Affiliations:** 1 Alligator Bioscience AB, Lund, Sweden; 2 Saromics Biostructures AB, Lund, Sweden; 3 Department of Immunotechnology, Lund University, Lund, Sweden

**Keywords:** 4-1BB, CD137, PD-1, Immunotherapy, Antibody, T cell activation

## Abstract

**Background:**

4-1BB (CD137) is a co-stimulatory receptor highly expressed on tumor reactive effector T cells and NK cells, which upon stimulation prolongs persistence of tumor reactive effector T and NK cells within the tumor and induces long-lived memory T cells. 4-1BB agonistic antibodies have been shown to induce strong anti-tumor effects that synergize with immune checkpoint inhibitors. The first generation of 4-1BB agonists was, however, hampered by dose-limiting toxicities resulting in suboptimal dose levels or poor agonistic activity.

**Methods:**

ATOR-1017 (evunzekibart), a second-generation Fc-gamma receptor conditional 4-1BB agonist in IgG4 format, was designed to overcome the limitations of the first generation of 4-1BB agonists, providing strong agonistic effect while minimizing systemic immune activation and risk of hepatoxicity. The epitope of ATOR-1017 was determined by X-ray crystallography, and the functional activity was assessed in vitro and in vivo as monotherapy or in combination with anti-PD1.

**Results:**

ATOR-1017 binds to a unique epitope on 4-1BB enabling ATOR-1017 to activate T cells, including cells with an exhausted phenotype, and NK cells, in a cross-linking dependent, FcγR-conditional, manner. This translated into a tumor-directed and potent anti-tumor therapeutic effect in vivo, which was further enhanced with anti-PD-1 treatment.

**Conclusions:**

These preclinical data demonstrate a strong safety profile of ATOR-1017, together with its potent therapeutic effect as monotherapy and in combination with anti-PD1, supporting further clinical development of ATOR-1017.

**Supplementary Information:**

The online version contains supplementary material available at 10.1007/s00262-023-03548-7.

## Introduction

Immunotherapy using approved immune checkpoint inhibitors (ICI) has firmly established immuno-oncology as the fourth pillar of cancer therapy. Still, not all patients respond to ICI for multiple reasons, including absence or exhaustion of existing tumor-infiltrating lymphocytes, subverting their anti-tumoral properties. There is a need for improvement of current cancer immunotherapies by combining multiple immunomodulatory targeting regimens and developing novel therapies. Immunostimulatory antibodies targeting co-stimulatory receptors such as 4-1BB have been shown in preclinical models to induce synergistic effects with ICI, for example programmed cell death protein-1 (PD-1) [1, 2], and with radiotherapy [3] or chemotherapy [4].

4-1BB (CD137, TNFRSF9) is a co-stimulatory receptor transiently expressed on various immune cells, primarily on effector T cells upon antigen recognition through their T-cell receptor, but also on regulatory T cells (Treg) and natural killer (NK) cells [5, 6]. Importantly, 4-1BB is highly expressed on tumor infiltrating CD8 + T cells, cells with the capacity to specifically recognize and kill tumor cells, while 4-1BB expression on circulating T cells is low [7–9]. More specifically, 4-1BB is expressed on exhausted CD8 + T cells within the tumor microenvironment. These cells are tumor antigen-specific but have a dysfunctional effector function, with the capacity to restore their desired function and promote tumor regression upon anti-4-1BB therapy [10].

The natural ligand to 4-1BB, 4-1BB ligand (4-1BBL) is a trimer expressed on antigen-presenting cells [11]. Immune activation through 4-1BB depends on formation of superclusters of 4-1BB to induce downstream signaling [12]. Stimulation of 4-1BB on T cells supports proliferation, cytokine production, cytolytic effector functions, reduced cell death, increased survival and enhanced memory differentiation [13, 14]. On NK cells, 4-1BB ligation increases cytokine release and cytolytic activity leading to improved anti-tumor responses [6, 15].

Several studies in experimental tumor models have demonstrated potent induction of tumor immunity by treatment with agonistic 4-1BB antibodies [16–18]. The efficacy and safety of 4-1BB antibodies, and other TNFR superfamily agonists, is affected by several factors such as binding affinity, binding epitope, isotype subclass and ability to crosslink with Fc-gamma receptors (FcγRs) [17, 19, 20]. The majority of monoclonal 4-1BB agonists are designed to be conditional agonists and utilize the crosslinking capacity of FcγRs to induce 4-1BB superclustering and functional activity [21]. Their FcγR-conditional activity depends on epitope, choice of Fc-domain and affinity [17]. The other category of 4-1BB agonists is FcγR-non-conditional agonists, which induces 4-1BB activation also in the absence of FcγR engagement, based on its binding epitope and/or Fc-domain. Urelumab, an FcγR-non-conditional first generation 4-1BB agonist, is a potent agonist but has been shown to induce clinical toxicities including hepatoxicity in some patients at doses > 1 mg/kg [22, 23]. Another first generation FcγR-conditional 4-1BB agonist, utomilumab, was designed with an IgG2, where the combination of binding epitope and Fc provided good tolerability with few adverse events and no dose-limiting toxicity reported for doses up to 10 mg/kg, however, utomilumab is weakly agonistic compared with urelumab [19, 24–26].

ATOR-1017 (evunzekibart) was designed to overcome the limitations of the first generation of 4-1BB agonists, providing a potent agonistic effect while improving tolerability by minimizing systemic immune activation. The preclinical data presented herein describes a unique binding epitope of ATOR-1017 and the ability of ATOR-1017 to FcγR-conditionally activate T cells and NK cells, which translates into a tumor directed and potent anti-tumor therapeutic effect in vivo that is enhanced in combination with anti-PD-1. Toxicity studies in non-human primates (NHP) demonstrated a favorable safety profile of ATOR-1017 and supports further clinical development.

## Material and methods

### Cell lines and primary cells

Cell lines were purchased (authenticated by vendor), mycoplasma tested, cultured according to supplier´s instructions, and used within 10 weeks of culture (≤ 20 passages). Chinese hamster ovary (CHO-K1, ATCC) cells were stably transfected with plasmids (pcDNA3.1 Amp/G418 vector, GenScript) with genes encoding human or cynomolgus 4-1BB, human Fc receptors: FcγRI, FcγRIIa R131, FcγRIIa H131 and FcγRIIb or CHO-pcDNA (mock transfected CHO-cells) using lipofectamine 3000 (Thermo Fisher Scientific). Single cell clones were sorted using flow cytometry (FACS Aria, BD Biosciences) or generated by limited dilution.

Human peripheral blood mononuclear cells (PBMC) were enriched from leucocytes concentrate obtained from healthy donors (Skåne University Hospital, Sweden). Cynomolgus PBMC were prepared from whole blood from cynomolgus macaques (Silabe). Primary CD8 + T cells were enriched from PBMC using human or non-human primate CD8 + T Cell Isolation Kits (Miltenyi Biotec), NK cells by negative selection (EasySep™ Human NK Cell Isolation Kit, StemCell Technologies), CD4 + T cells by negative selection (human CD4 T cell isolation kit, Miltenyi Biotec) and monocytic cells by positive selection (human CD14 microbeads, Miltenyi Biotec).

MC38 mouse colorectal carcinoma cell line was provided by Biocytogen (purchaser: Shunran Shanghai Biological Technology Co).

### Mice

For all in vivo and ex vivo flow cytometry studies, female homozygous humanized 4-1BB knock in mice (hu4-1BB KI, strain C57BL/6-*Tnfrsf9*^*tm1(TNFRSF9)*^/Bcgen, Beijing Biocytogen) were used where the human 4-1BB extracellular domain is knocked in to replace the mouse 4-1BB extracellular domain (exon 3–8).

### Generation of antibodies

ATOR-1017 and a human IgG4 isotype control (anti-GFP) were isolated from Alligator Bioscience proprietary antibody library ALLIGATOR-GOLD®. Specific 4-1BB binders were identified, screened, and further evaluated in IgG4 format (S228P) [27]. The selected anti-4-1BB binder was optimized for developability using selection and screening from designed libraries and FIND technology [28].

ATOR-1017 was expressed from a stable CHO DG44 cell line (Sartorius) and purified by MabSelect MabSuRe Protein A. The human IgG4 isotype, an ATOR-1017 binder with an IgG1 isotype and analogues of urelumab, utomilumab, ADG106 and CTX471 [19, 29, 30] were all expressed by transient transfection of ExpiCHO-S cells (Gibco/Thermo Fisher Scientific). Biotinylated ATOR-1017 was generated using EZ-link sulfo-NHS-LC-Biotin kit (#21,335, Thermo Fisher Scientific).

### Binding to 4-1BB

Binding affinity to human or cynomolgus 4-1BB (human CD137-His and cynomolgus 4-1BB/TNFRSF9-His, Acro) and to human and mouse FcγR and FcRn (Supplementary Table S1) was determined using Octet Red96 Bio-Layer Interferometry (BLI) platform (ForteBio).

CHO-K1 cells transfected to stably express human 4-1BB (CHO-hu4-1BB), cynomolgus 4-1BB (CHO-cy4-1BB) or CHO-pcDNA were incubated with serially diluted ATOR-1017 and binding was detected with goat α-hIg Fc-PE (Jackson ImmunoResearch). Mean fluorescence intensity (MFI) was determined with flow cytometry (FACSverse, BD Biosciences) and analyzed using FlowJo software.

Binding of ATOR-1017 to human and cynomolgus 4-1BB on activated primary immune cells was assessed on freshly isolated PBMC. PBMC were activated 48 h with anti-CD3 (cynomolgus anti-CD3, clone FN-18, Invitrogen or human anti-CD3, clone OKT3, eBioscience), Fc-blocked (Beriglobin, CSL Behring) and then incubated with serially diluted biotinylated ATOR-1017 followed by streptavidin-APC, CD3-PECy7(SP34-2), CD8-APC-H7(SK1) and fixable viability stain 510 (BD Pharmingen) and analyzed with flow cytometry.

### 4-1BB ligand blocking

CHO-hu4-1BB cells were pre-incubated 1 h with serially diluted ATOR-1017 or IgG4 isotype before addition of a fixed concentration of human 4-1BBL (Ancell). Bound 4-1BBL was detected with anti-FLAG-APC (Cell signaling technology) using flow cytometry. The relative loss of binding of 4-1BBL (%) normalized to maximal 4-1BBL binding was calculated.

### Complex formation and crystal structure determination of 4-1BB and ATOR-1077

The crystal structure of the complex between human 4-1BB and ATOR-1017 in single-chain variable fragment (scFv) format was determined at 3.1 Å and has been deposited in the protein data bank with PDB ID 8OZ3. The complex was generated by mixing a 4-1BB variant produced and purified as described in Nelson et al. 2023 [31] and ATOR-1017scFv produced and purified from Top10 bacterial cultures as depicted in Supplementary Figure S1 at a molar ratio of 1:1.3 and purified on a Superdex 75 column. Crystals of 4-1BB: ATOR-1017scFv complex at 12.5 mg/ml in 10 mM HEPES pH7.5, 150 mM NaCl generated after seeding, as depicted in Supplementary Figure S2, as a multi-needle crystal bunch. A single needle was removed from the bunch and used for data collection at 100 K at the BioMAX beamline, MAX IV, Lund, Sweden (λ = 0.992 Å) equipped with a Pilatus 6 M-F detector. The data set was collected using a helical scan starting at one part of the thin rod and ending at another part of the rod using exposure time of 0.03 s and an oscillation of 0.1° per image collecting 360° in total. The data were processed in XDS [32] and Aimless [33] in space group P22_1_2_1_. The structure was determined using molecular replacement with Phaser [34] using as templates a homologous Fab (PDB: 2UZI) and a published 4-1BB structure [31] (PDB 7YXU). The structure was refined in Refmac5 [35] and model building was carried out in Coot [36]. The final model includes ammino acids 26–161 for both copies of 4-1BB, amino acids 2–118 for the heavy chains and amino acids 2–110 for the light chains of the variable domains of ATOR-1017scFv. In addition, 10 water molecules have been modelled and refined. The final R/R_free_ values obtained are 0.205/0.271 (Supplementary Table S2). Epitope analysis was done using the CONTACT software in the CCP4 suite program [37].

### 4-1BB Bioassay and ADCC reporter assays

FcγR-conditional 4-1BB activation was demonstrated with a 4-1BB Bioassay (Promega). Bioassay effector cells were co-cultured 6 h with FcγRI-CHO cells and serially diluted ATOR-1017, before luminescence was measured on a FLUOStar OPTIMA microplate reader (BMG Labtech).

Antibody-dependent cell-mediated cytotoxicity (ADCC) function was assessed using an ADCC reporter Bioassay (Promega). Antibodies were serially diluted and incubated with FcγRIIIa-V158 effector cells and CHO-hu4-1BB target cells (5:1 ratio). Effector cell engagement was measured as luminescence.

### CD8 + T cell activation assay

UV-irradiated FcγR-CHO (FcγRI, FcγRIIb and FcγRIIa R and H), or CHO-pcDNA cells (0.2 × 10^6^ cells/well) were cultured overnight. The following day, 4-1BB antibodies were pre-incubated with CHO cells, prior to the addition of anti-CD3 coated beads (Dynal M-450 tosyl activated, Invitrogen) and human (or cynomolgus) CD8 + T cells (0.1 × 10^6^ cells/well, 1:1 bead to effector cell ratio). Cell culture supernatants were harvested and IFNγ, granzyme B and perforin levels were measured by ELISA (Mabtech and BD Biosciences) or Human TH1/TH2 10-Plex Tissue Culture Kit (MSD). CD8 + T cells were stained with CD3-FITC(UCHT-1), CD25-BV421(2A3), CD107a-APC-Cy7(H4A3), CD137-PE(4B4-1), CD278-PerCP-Cy5.5(DX29) and live stain FVS510 (BD Biosciences) and analyzed by flow cytometry.

Human NK cell (0.5 × 10^5^ cells/well), pre-stimulated with IL-2 (10 ng/mL, 2.1E4 IU/µg, R&D) for 24 h, were co-cultured 24–72 h with FcγRI-CHO cells (0.1 × 10^6^ cells/well), serially diluted ATOR-1017 or IgG4 isotype and IL-2. IFNγ and granzyme B was measured in supernatants by ELISA.

### Mixed lymphocyte reactions (MLR)

MLR assay was performed with monocyte derived dendritic cells (moDCs) co-cultured with allogeneic exhausted T cells. To generate exhausted T cells, CD4 + T cells were expanded for 7 days with Dynabeads™ Human T-Activator CD3/CD28 (1:1 bead to cell ratio; Invitrogen) replaced every second day. After 7 days, Dynabeads were removed and CD4 + T cells were rested overnight. MoDCs were generated from CD14 + monocytic cells in the presence of IL-4 and GM-CSF (Miltenyi Biotech) for 5 days and then matured for 24 h using a cocktail of Il-1β, IL-6, TNF-α (Miltenyi Biotech) and PGE2 (Merck Millipore). Mature moDCs and exhausted CD4 + T cells were co-cultured (ratio 1:10) with serially diluted Opdivo® and ATOR-1017 crosslinked with F(ab)2 anti-Ig (5:1 molar ratio to mAb) for 7 days, when IFNγ in supernatants was analyzed by ELISA. Exhausted CD4 + T cells and mature moDCs phenotypes was confirmed with flow cytometric staining: CD4-PerCpCy5.5(RPA-T4), PD-1-PE(MIH4), CD14-PerCpCy5.5(MφP9), CD86-FITC(2331FUN-1) and HLADR-BV510(G46-6) from BD Biosciences; LAG-3-FITC(11C3C65), TIM-3-BV421(F38-2E2), CTLA-4-BV421(BNI3), CD137-PECy7(4B4-1) (BioLegend); TOX-PE(REA473) (Miltenyi Biotech) and PD-1-APC(MIH4) (eBioscience).

### Tumor growth assessment and flow cytometry analysis

Hu4-1BB KI mice (6–10 weeks) were injected subcutaneously (sc) on the right hind flank with MC38 cells (0.5 × 10^6^). Seven days after later MC38-tumor-bearing mice were randomly enrolled into different treatment groups (10 mice/group) based on tumor size and treated twice weekly intraperitoneally (ip) with ATOR-1017, human IgG4(S228P) isotype control (CrownVivo), anti-PD-1 (RMPI-14, BioXcell) or ATOR-1017 + anti-PD-1 for three weeks. Tumor volume and body weight were measured, and survival was monitored. Complete responder mice (> 100 days) were re-challenged with MC38 cells injected sc in the left hind flank. Animals were euthanized when the ethical humane endpoints were reached, including tumor volume exceeding 3 cm^3^, tumor ulceration or affected health.

For flow cytometry analysis, MC38 bearing mice, randomly enrolled into two groups (8 mice/group) when the average tumor volume reached 220 mm^3^, received either human IgG4(S228P) isotype control, or ATOR-1017 twice weekly for four doses. 48 h after last treatment, tumors and spleens were harvested and weighed. Tumors were cut into small pieces and enzymatically digested with Tissue dissociation kit (Miltenyi Biotec). The single-cell suspensions were Fc blocked before staining with the following anti-mouse antibodies: CD16/32 (clone 93), CD45-BV421(30-F11), CD3-AF488(17A2), CD4-BV510(RM4-5), CD8a-BV711(53–6.7) (Biolegend); NK1.1-PE-Cy7(PK136) (BD Pharmingen); hCD137-PE(4B4-1), Foxp3-APC(FJK-16 s) (eBioscience). Zombie NIR™ (Biolegend) was used to stain non-viable cells. Relative numbers (%) and absolute counts were determined with flow cytometry (Attune NxT, Thermo Fisher) and analyzed using FlowJo software.

### Preclinical safety assessment

Cynomolgus macaques (*n* = 24) were given five, once‐weekly, 1‐hour IV doses of ATOR-1017 (5, 15, or 50 mg/kg) or vehicle control consisting of three males and females per treatment group. Standard safety parameters and endpoints were evaluated. At study termination, a complete necropsy examination was performed. Pharmacokinetic parameters were evaluated on days 1 and 22. ATOR-1017 serum concentration was determined by ELISA (VarioskanFlash, Thermo Fisher Scientific) and data collection and analysis was performed using SkanIt and Watson software (Thermo Fisher Scientific).

### Statistics

GraphPad Prism 8 (GraphPad Software, Inc) was used to generate graphs and statistics. Where indicated, the difference between groups was evaluated using non-parametric Mann–Whitney *U,* two-tailed test. Survival curves were analyzed using the Kaplan Meier method and compared using Log-rank (Mantel-Cox). PK parameters were estimated using Phoenix (WinNonlin) pharmacokinetic software using a non‐compartmental analysis approach.

## Results

### ATOR-1017 binds to a unique epitope on 4-1BB

The parental lead anti-4-1BB binding domain obtained from the human scFv antibody library ALLIGATOR-GOLD® was optimized for stability and developability in the IgG4 (S228P) format. The optimized antibody sequence displayed reduced aggregation propensity and hydrophobic patches relative to the parental clone.

The affinity of ATOR-1017 to human and cynomolgus 4-1BB was determined, using BLI, to 0.1 nM to human 4-1BB and 0.2 nM to cynomolgus 4-1BB. Binding to human and mouse FcγR and FcRn was confirmed and comparable with an IgG4 control antibody (Supplementary Table S1).

**Fig. 1:**
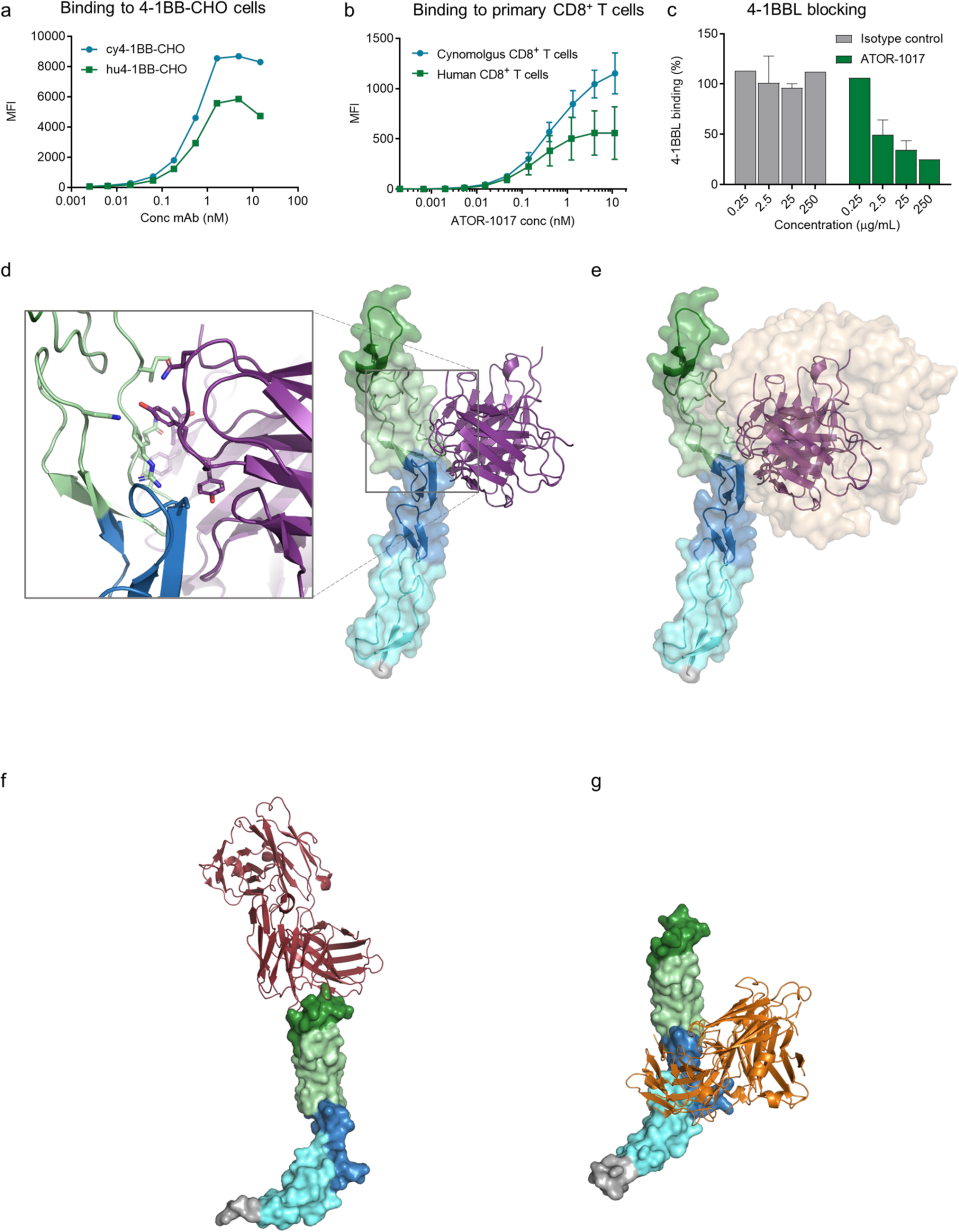
ATOR-1017 and 4-1BB binding characterization** a**, Binding of ATOR-1017 to CHO cells expressing either human or cynomolgus 4-1BB (hu4-1BB-CHO or cy4-1BB-CHO). CHO cell lines were incubated with serially diluted ATOR-1017 and binding was detected with anti-hIg Fc-PE using flow cytometry. Binding curves (MFI) from one representative out of two experiments is shown. **b**, Binding of ATOR-1017 to 4-1BB on primary human and cynomolgus CD8 + T cells. Primary human or cynomolgus PBMCs were stimulated for 48 h with anti-CD3. Binding of serially diluted biotinylated ATOR-1017 was detected with streptavidin-APC followed by staining for cell surface markers for T cells and analyzed for binding using flow cytometry. Binding was evaluated on cells gated on viable CD3 + CD8 + single cells. MFI of pooled data from 2 experiments (*n* = 6–8/group) is presented. **c,** Ability of ATOR-1017 to block 4-1BBL binding. hu4-1BB-CHO cells were pre-incubated 1 h with serially diluted ATOR-1017 or IgG4 isotype control (anti-GFP) before addition of 4-1BBL at a fixed concentration of 7 µg/ml. After 30 min co-incubation, bound 4-1BBL was detected with anti-FLAG-APC by flow cytometry. Data was normalized and plotted as % of maximal 4-1BBL binding in the absence of antibody. **d-g**, Crystal structures for 4-1BB complexed with **d**, ATOR-1017 scFv (PDB ID 8OZ3), **e**, 4-1BB trimeric ligand (PDB ID 6MGP), **f**, urelumab (PDB ID 6MHR) and **g**, utomilumab (PDB ID 6MI2). **d**, ATOR-1017 binds to domain 2 (pale green) and domain 3 (marine blue) on the 4-1BB receptor and **e,** overlaps with the binding site for the trimer 4-1BB ligand (sand). ATOR-1017 binding site differs substantially from **f**, urelumab (red) and **g**, utomilumab (orange) binding sites

Binding potency of ATOR-1017 to human or cynomolgus 4-1BB expressed either on transfected CHO cells (Fig. 1a) or on activated primary CD8 + T cells (Fig. 1b) was similar. EC50 to hu4-1BB-CHO was determined to 0.5 nM (0.3–0.5) vs. 0.5 nM (0.4–0.6) to cy4-1BB-CHO, and 0.3 nM to human CD8 + T cells vs 0.6 nM to cynomolgus CD8 + T cells.

In a 4-1BB ligand blocking experiment it was demonstrated that ATOR-1017 blocks the 4-1BBL binding to 4-1BB (Fig. 1c). This was further confirmed by X-ray crystallography of ATOR-1017 in scFv format complexed with hu4-1BB, which demonstrated that ATOR-1017 interacts with residues on domain 2 and 3 of 4-1BB (Fig. 1d, and Supplementary Table 3) forming a binding epitope for ATOR-1017 that overlaps with that of 4-1BBL (Fig. 1e) but not with urelumab (Fig. 1f) or utomilumab (Fig. 1g).

### ATOR-1017 induce FcγR-crosslinking dependent 4-1BB activity, while avoiding ADCC function for improved safety and efficacy

**Fig. 2:**
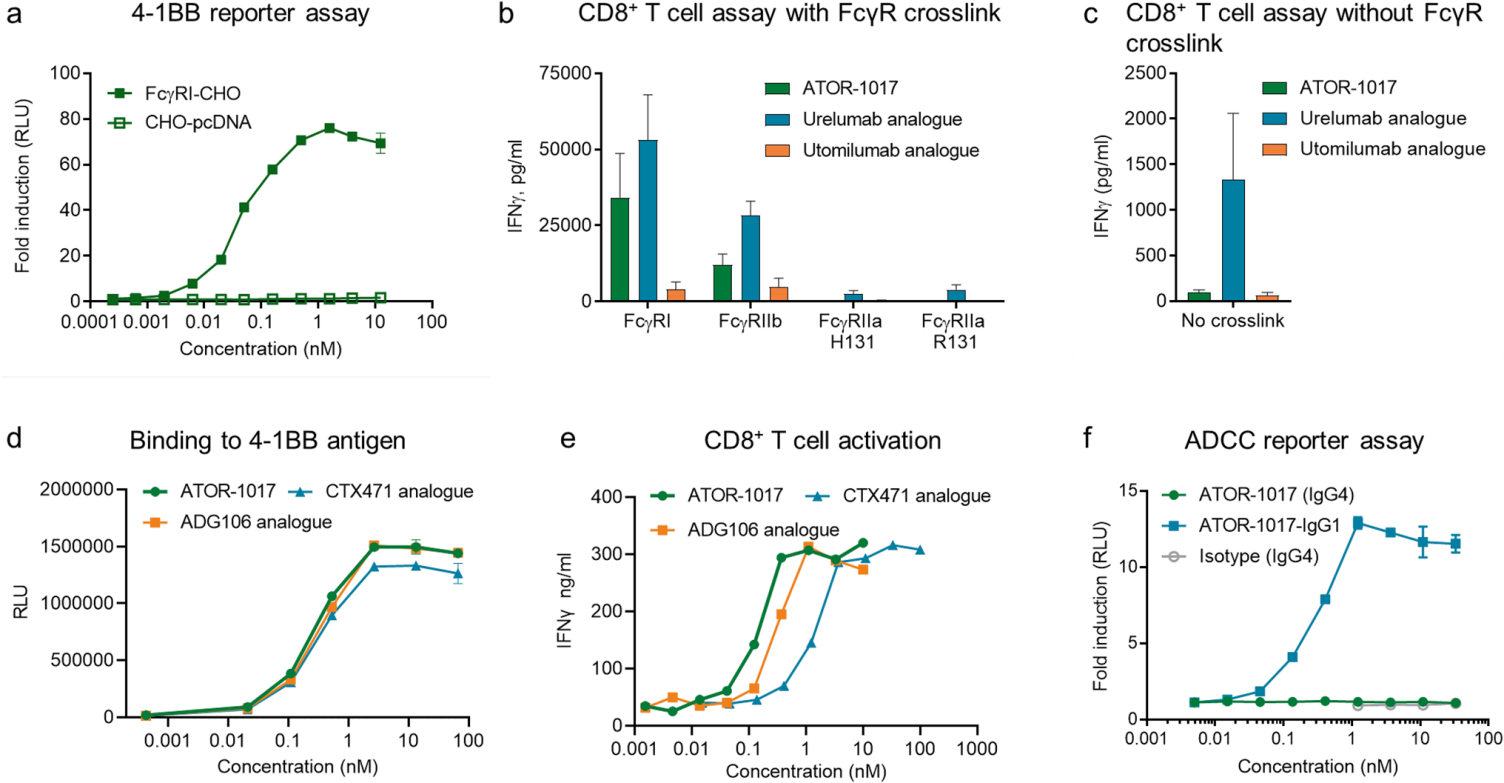
Functional characterization of FcγR dependent agonistic activity of ATOR-1017 **a**, 4-1BB Bioassay reporter cells were stimulated with serially diluted ATOR-1017 in the presence of FcγRI-CHOI or CHO-pcDNA cells for 6 h before measuring luminescence. Mean and SD from duplicate samples from one representative experiment is shown. **b-c**, Activation of human CD8 + T cells. **b**, UV-irradiated CHO cells expressing different FcγRs (FcγRI, FcγRIIb and FcγRIIa) or **c,** mock-transfected CHO cells (0.2 × 10^6^ cells/well) were plated overnight. CD8 + T cells (*n* = 6–10 donors/group) were activated with anti-CD3 coated beads. ATOR-1017 and analogues of urelumab (IgG4) and utomilumab (IgG2) were pre-incubated with the CHO cells, prior to the addition of CD3-coated beads and CD8 + T cells (0.1 × 10^6^ cells/well) at a ratio 1:1 for beads and effector cells. After 72 h incubation at 37 °C, cell culture supernatants were harvested and IFNγ levels were measured by ELISA. Mean and SD from three experiments is shown. **d**, Binding to 4-1BB antigen by ATOR-1017 and ADG106 and CTX471, two 4-1BB IgG4 analogue mAbs currently in clinical development, was determined using ELISA. Mean and SD from duplicate samples from one representative experiment out of two is shown. **e**, Activation of human CD8 + T cells with ATOR-1017 and analogues of ADG106 and CTX471 in co-cultures with FcγRI-CHO cells. After 72 h, cell culture supernatants were harvested and IFNγ levels were measured by ELISA. Mean from one representative donor is shown. **f**, No induction of ADCC by ATOR-1017 in an ADCC reporter assay. Antibodies including ATOR-1017, ATOR-1017-binder with an IgG1 Fc and IgG4 isotype control (anti-GFP) were incubated for 6 h with FcγRIIIa-V158 expressing effector cells and hu4-1BB-CHO target cells. Effector cell engagement induced by the antibodies was quantified as production of luciferase and measured as luminescence. Mean and SD of fold-change to background signal from one representative experiment is presented

The activity of ATOR-1017 in a 4-1BB/NFΚB reporter bioassay was dependent on FcγR engagement as there was no activation in the co-cultures with CHO-pcDNA cells (Fig. 2a). Comparable results were obtained in a primary CD8 + T cell activation assay, were ATOR-1017 FcγR-conditional 4-1BB activation was compared with two analogues of the first generation of 4-1BB antibodies, urelumab (IgG4) and utomilumab (IgG2). In the presence of FcγR crosslinking, ATOR-1017 and urelumab induced potent T cell activation (as measured by an increase in IFNγ release), whereas utomilumab, having an IgG2 format, only induced minor T cell activity (Fig. 2b). In the absence of crosslinking by FcγRs, ATOR-1017 and utomilumab did not activate T cells, whereas urelumab showed 4-1BB activity also without crosslinking (Fig. 2c). The presence of soluble IgG reduced the activity of ATOR-1017 in a dose dependent manner (Supplementary Figure S3).

Moreover, ATOR-1017 had similar binding potency compared to ADG106 and CTX471 4-1BB IgG4 mAbs currently in clinical development (Fig. 2d) but displayed a superior potency in the CD8 + T cell activation assay (Fig. 2e).

In an ADCC reporter assay system, ATOR-1017 (IgG4) had a low potential for inducing ADCC of 4-1BB expressing CHO-cells (Fig. 2f).

### ATOR-1017 activates both T cells and NK cells in vitro

**Fig. 3:**
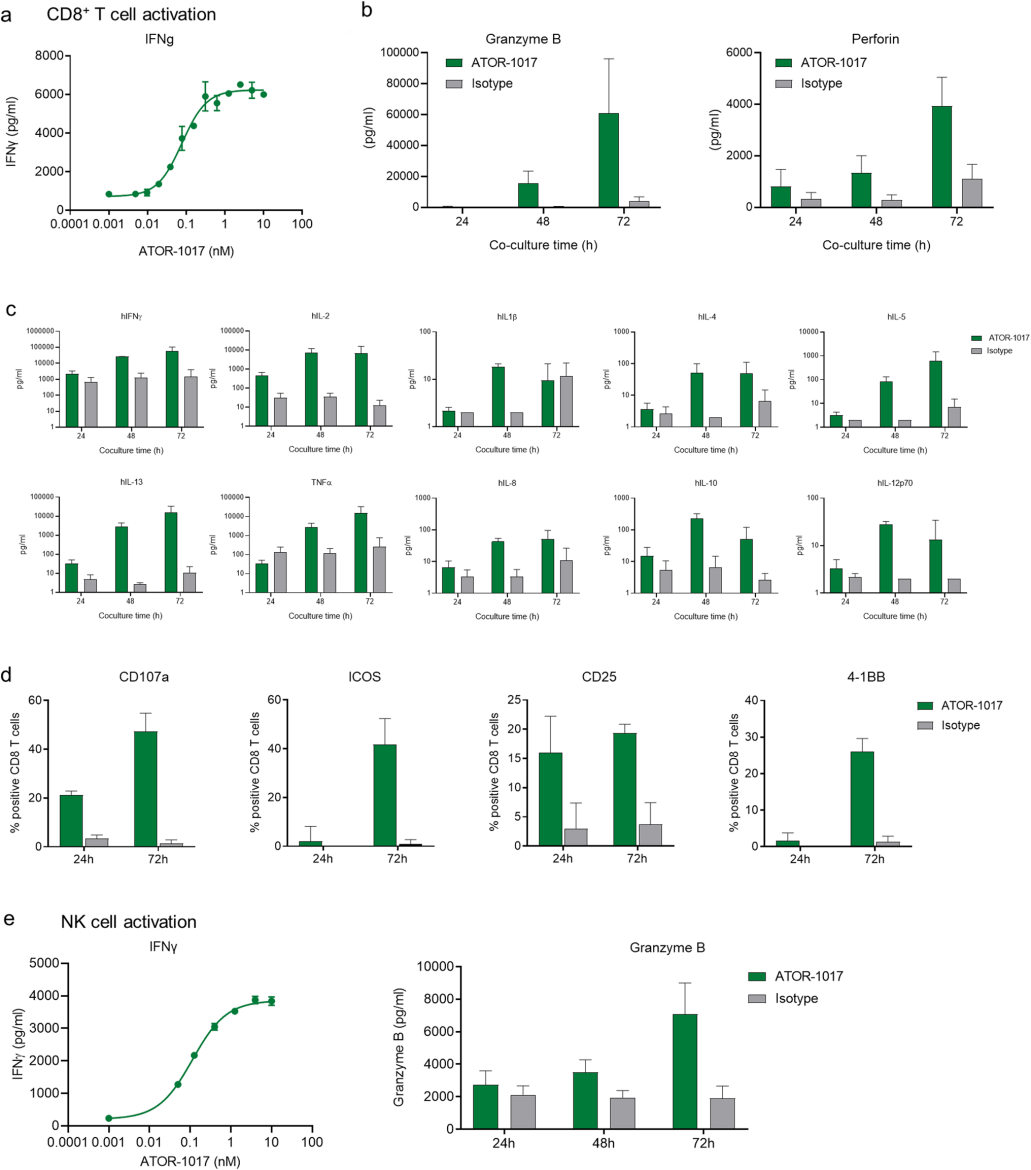
Potent activation of both T cells and NK cells **a-d**, Activation of CD8 + T cells with ATOR-1017. **a**, ATOR-1017 induces dose-dependent activation of human CD8 + T cells. UV-irradiated FcγRI-CHO cells (0.2 × 10^6^ cells/well) were cultured overnight. CD8 + T cells (*n* = 3–12 donors/group) were activated with sub-optimal concentration of anti-CD3 coated beads. ATOR-1017 or isotype control were pre-incubated with CHO cells, prior to the addition of αCD3 coated beads and CD8 + T cells (0.1 × 10^6^ cells/well) at a ratio 1:1 for beads and effector cells. Cell culture supernatants were harvested **a**, after 72 h to analyze levels of IFNγ (*n *= 12) and **b**, after 24, 48 and 72 h to analyze levels of granzyme B and perforin at 0.1 nM of ATOR-1017 (*n* = 3) with ELISA. **c**, Th1/Th2 cytokine production from CD8 + T cells activated with ATOR-1017. CD8 + T cells were stimulated with 0.1 nM ATOR-1017 or isotype control and anti-CD3 coated beads in co-cultures with FcγRI-CHO cells. After 24 h (*n* = 6), 48 h (*n* = 3) and 72 h (*n* = 12), levels of IFNγ, IL-2, IL-13, TNFa, IL-1b, IL-4, IL-5, IL-8, IL-10 and IL-12p70 from supernatants were analyzed. Mean ± SD from four experiments is shown. **d**, Flow cytometry of CD8 + T cells activated with ATOR-1017. Following 24 h or 72 h stimulation with ATOR-1017 or IgG4 isotype control in co-cultures with FcγRI cells, markers for activation (CD107a, ICOS, CD25 and 4-1BB) on CD8 + T cells were analyzed by flow cytometry. The percentage of positive cells of total CD8 + T cells (mean and SD, *n* = 3) is presented. **e**, ATOR-1017 induces dose-dependent activation of human NK cells. NK cells were pre-stimulated with IL-2 (10 ng/mL) overnight. UV-irradiated FcγRI-CHO cells (0.1 × 10^6^ cells/well) were plated overnight. The following day, serially diluted ATOR-1017 or isotype control, pre-stimulated NK cells (0.5 × 10^5^ cells/well) supplemented with Na-pyruvate (1 mM) and IL-2 (10 ng/mL) were added to the FcγRI-CHO cells. After 24–72 h incubation, cell culture supernatants were harvested for analyzation of levels of IFNγ (serially diluted ATOR-1017) and granzyme B (at 0.2 nM of ATOR-1017) by ELISA. Mean and SD from 3 to 6 representative donors are shown

ATOR-1017 induced an FcγR-conditional activation of CD8^+^ T cells as measured by IFNγ production (EC50 0.2 nM, Fig. 3a) as well as increases in granzyme B and perforin levels (Fig. 3b). In addition, ATOR-1017 induced strong increases in the production of Th1 cytokines such as IFNγ, IL-2, IL-13 and TNFα, and lower induction of IL-1B, IL-4, IL-5, IL-8, IL-10 and IL-12p70 (Fig. 3c). Moreover, CD8^+^ T cells activated with ATOR-1017 upregulated activation markers CD107a, ICOS, CD25 and 4-1BB (Fig. 3d). Further, ATOR-1017 induced FcγR-conditional activation of NK cells as measured by IFNγ (EC50: 0.1 nM) and increase in granzyme B (Fig. 3e).

### ATOR-1017 induces a tumor-directed immune activation resulting in a potent anti-tumor response in vivo

**Fig. 4:**
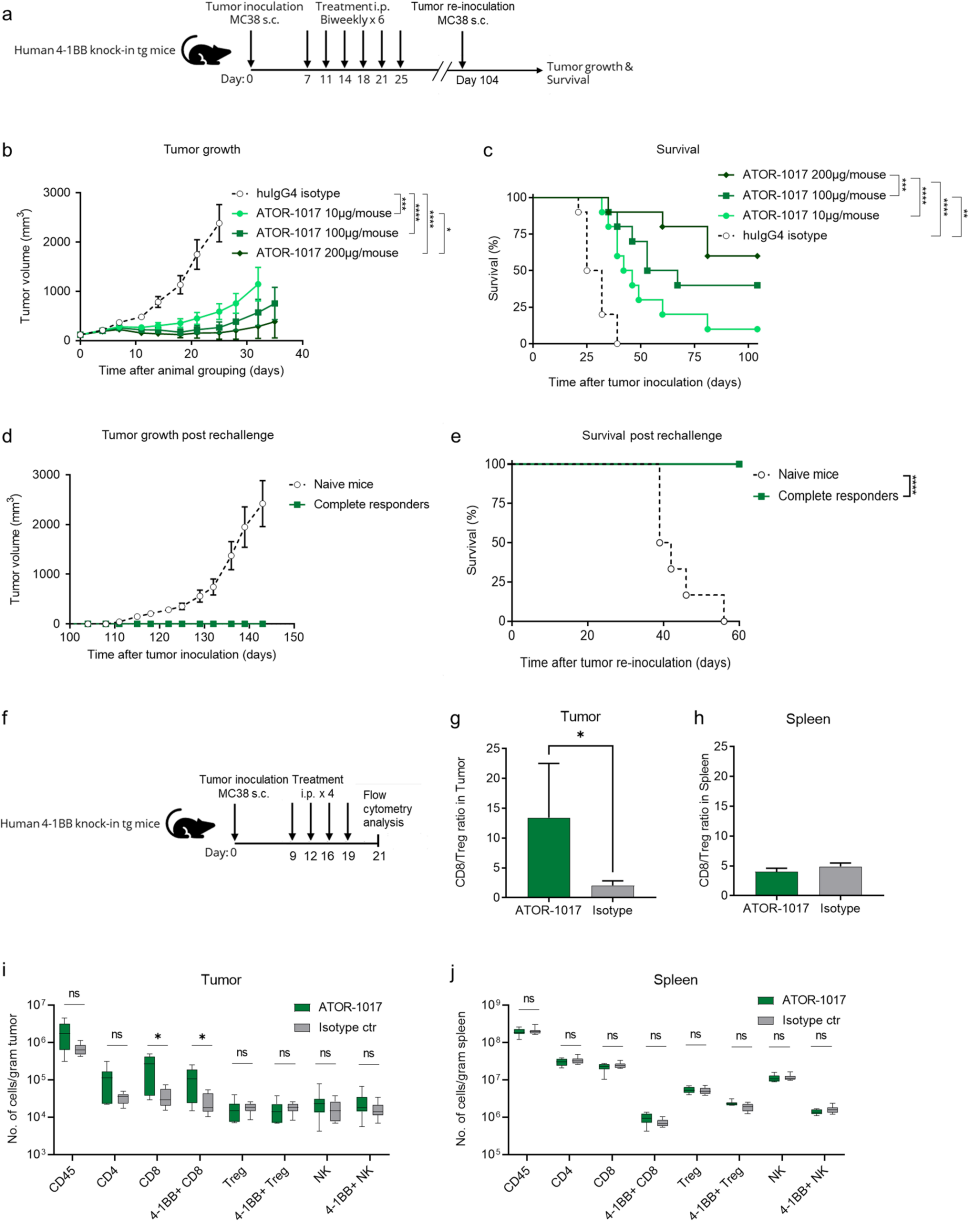
Anti-tumor activity and immune activation of ATOR-1017 in MC38 tumor-bearing homozygous human 4-1BB knock-in mice** a**, Experimental design for anti-tumor efficacy studies and rechallenge experiments. Six to ten weeks old hu4-1BB KI mice were injected subcutaneously (sc) on the right hind flank on day zero with MC38 cells (0.5 × 10^6^ cells). Seven days post tumor inoculation, mice (*n* = 10/group) were randomly distributed to four different treatment groups with the average tumor volume of 119 mm^3^ per treatment group. Intraperitoneal treatments with human IgG4 isotype control (100 µg/dose) or ATOR-1017 (10, 100, 200 µg/dose) were administered on days 7, 11, 14, 18, 21 and 25. After more than 100 days, complete responders (*n* = 11) were rechallenged with MC38 cells and their tumor growth were compared with naïve mice (*n* = 6). **b** and **d**, Tumor volume depicted as mean ± SEM. **c** and **e,** survival. (**, *p* < 0.01; ***, *p* < 0.001; ****, *p* < 0.0001). **f**, Experimental design for immune activation ex vivo studies. Immunological effects of ATOR-1017 or IgG4 isotype control in MC38 tumor-bearing human 4-1BB knock-in mice (*n* = 8). Nine days post tumor inoculation, mice were distributed into two treatment groups. Intraperitoneal treatments with huIgG4 (100 µg/dose) or ATOR-1017 (100 µg/dose) were administered on days 9, 12, 16 and 19, and ex vivo flow cytometry analysis was performed on spleen and tumor tissue day 21, 48 h after the last treatment. **g-h**, CD8 + /Treg ratio in **g**, tumor (*n* = 7) and **h**, spleen (*n* = 8). Statistical differences comparing ATOR-1017 to isotype were analyzed using Mann–Whitney, non-parametric 2-tailed t-test (*, *p* < 0.05). **i-j**, Number of lymphocytes per gram tissue in **i,** tumor (*n *= 7) and **j,** spleen (*n* = 8). The absolute number of lymphocytes assessed after flow cytometry was divided with the weight (grams) of tumor or spleen measured before tissue dissociation. Statistical differences comparing ATOR-1017 to isotype were analyzed using Mann–Whitney, non-parametric 2-tailed t-test (*, *p* < 0.05)

In a MC38 tumor model (Fig. 4a) ATOR-1017 induced a potent dose-dependent tumor growth control (Fig. 4b) and enhanced survival (Fig. 4c). Upon tumor rechallenge, tumors only developed in the naïve control mice and not in the complete responder mice, confirming induction of a long-term immunological memory against MC38 following ATOR-1017 treatment (Fig. 4d, e).

In a similar set-up (Fig. 4f) the immune stimulatory effect of ATOR-1017 was demonstrated to be tumor-directed resulting in an increased ratio of CD8 + T cells vs Tregs in the tumor (Fig. 4g) but not in the periphery (in the spleen, Fig. 4h). A significant increase in the number of CD8 + T cells and 4-1BB expressing CD8 + T cells was detected in ATOR-1017 vs. isotype control treated mice in the tumor (Fig. 4i), but not in the spleen (Fig. 4j).

**Fig. 5:**
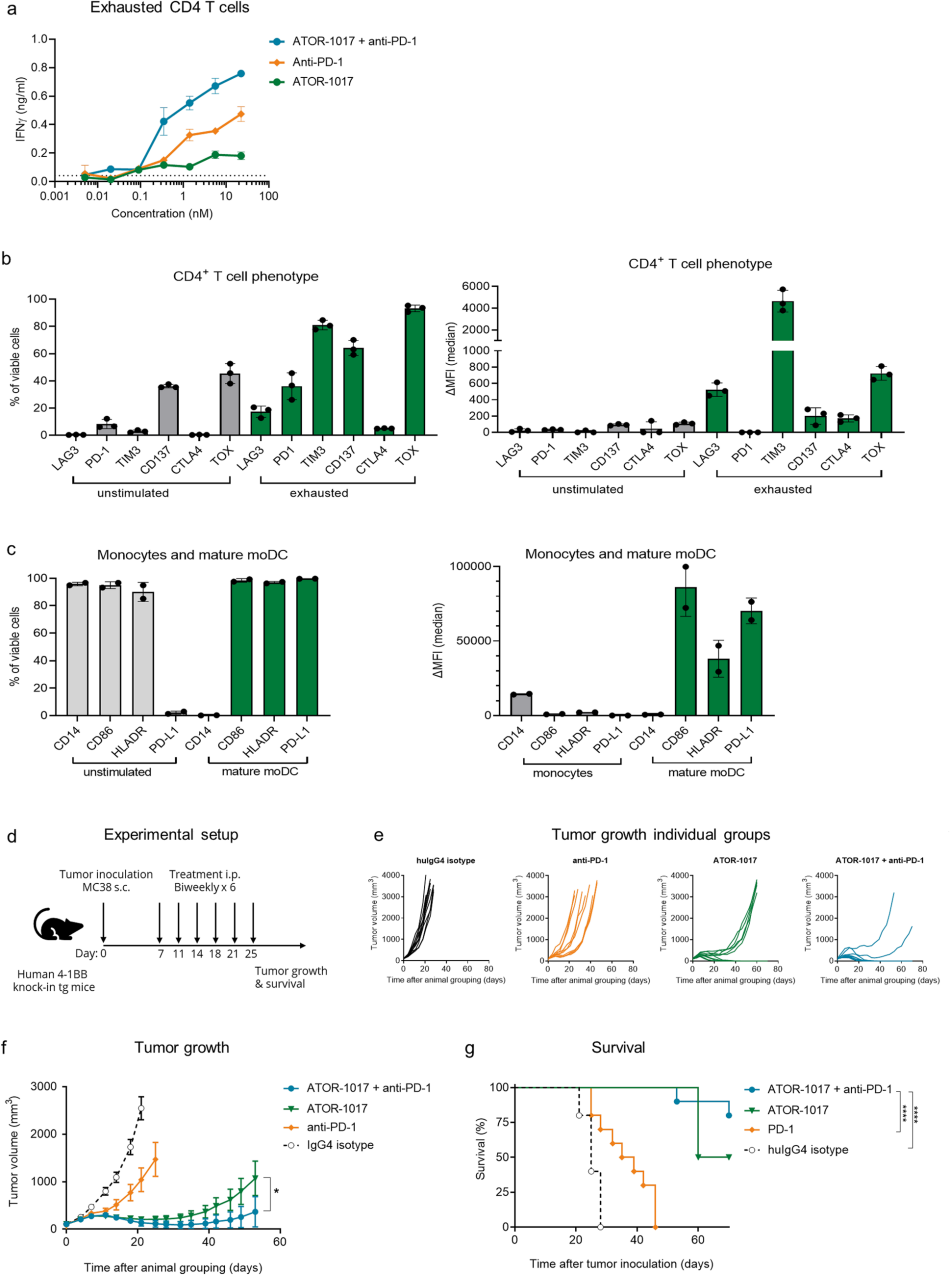
ATOR-1017 in combination with anti-PD-1 improves activation of T cells in MLR assays in vitro and improves anti-tumor activity in vivo **a**, ATOR-1017 was evaluated in combination with anti-PD-1 antibody Opdivo® in MLR assays using allogeneic exhausted human primary CD4 + T cells and mature moDCs. CD4 + T cells with an exhausted phenotype were expanded for 7 days with DynaBeads and then rested overnight in medium. Titrations of ATOR-1017 and anti-PD-1 in the presence of F(ab)2 anti-Ig crosslinker (at a 5:1 molar ratio to ab) were used in cultures with a 1:10 mix of mature Mo-DC cells and exhausted CD4 + T cells for 7 days before cell culture supernatants were harvested for analyzing IFNγ levels by ELISA. **b-c**, Phenotypic characterization of stimulated CD4 + T cells and moDC cells. Activation markers on unstimulated CD4 + T cells (day -2) or exhausted CD4 + T cells (day 6) and unstimulated monocytes (day 0) or Mo-DC cells (day 6) were measured by flow cytometry. CD4 + T cells and monocytes were analyzed as percentage of positive cells of viable cells (left column) and ΔMFI of the positive fraction with blank subtracted (right column). **d**, Experimental setup for anti-tumor efficacy studies of ATOR-1017 in combination with mouse anti-PD1 mAb. Seven days post tumor cell inoculation, MC38 colon carcinoma bearing homozygous human 4-1BB knock-in mice (n = 10) were randomly distributed into different treatment groups based on tumor size, with a mean tumor volume of 119 mm^3^ for all groups on day 7. Intraperitoneal treatments with ATOR-1017 (100 µg/dose), mouse anti-PD-1 (100 µg/dose, RMPI-14) or human IgG4 (S228P) isotype control (100 µg/dose), were given on days 7, 11, 14, 18, 21 and 25. Tumor volume and survival was assessed over time and depicted as **e,** individual tumor volume measurements for each mouse per treatment group or **f**, mean ± SEM per treatment group and **g**, Survival. Statistical differences were analyzed using Mann–Whitney, non-parametric t-test for tumor growth (**f**) and Kaplan Meier, Log-Rank for survival (**g**) (*, *p* < 0.05; **, *p* < 0.01; ***, *p* < 0.001; ****, *p* < 0.0001)

The ability of ATOR-1017 in combination with anti-PD-1 to rescue exhausted T cells was investigated in MLR assays. The results demonstrated synergistically improved re-activation of CD4 + T cells with an exhausted phenotype (Fig. 5a) expressing typical exhaustion markers (Fig. 5b). Characterization of the matured moDCs were also included in the analysis (Fig. 5c). Furthermore, combination treatment with ATOR-1017 and anti-PD-1 treatment resulted in enhanced anti-tumor efficacy in the MC38 tumor model (Fig. 5d-g).

### Preclinical safety evaluation

**Fig. 6:**
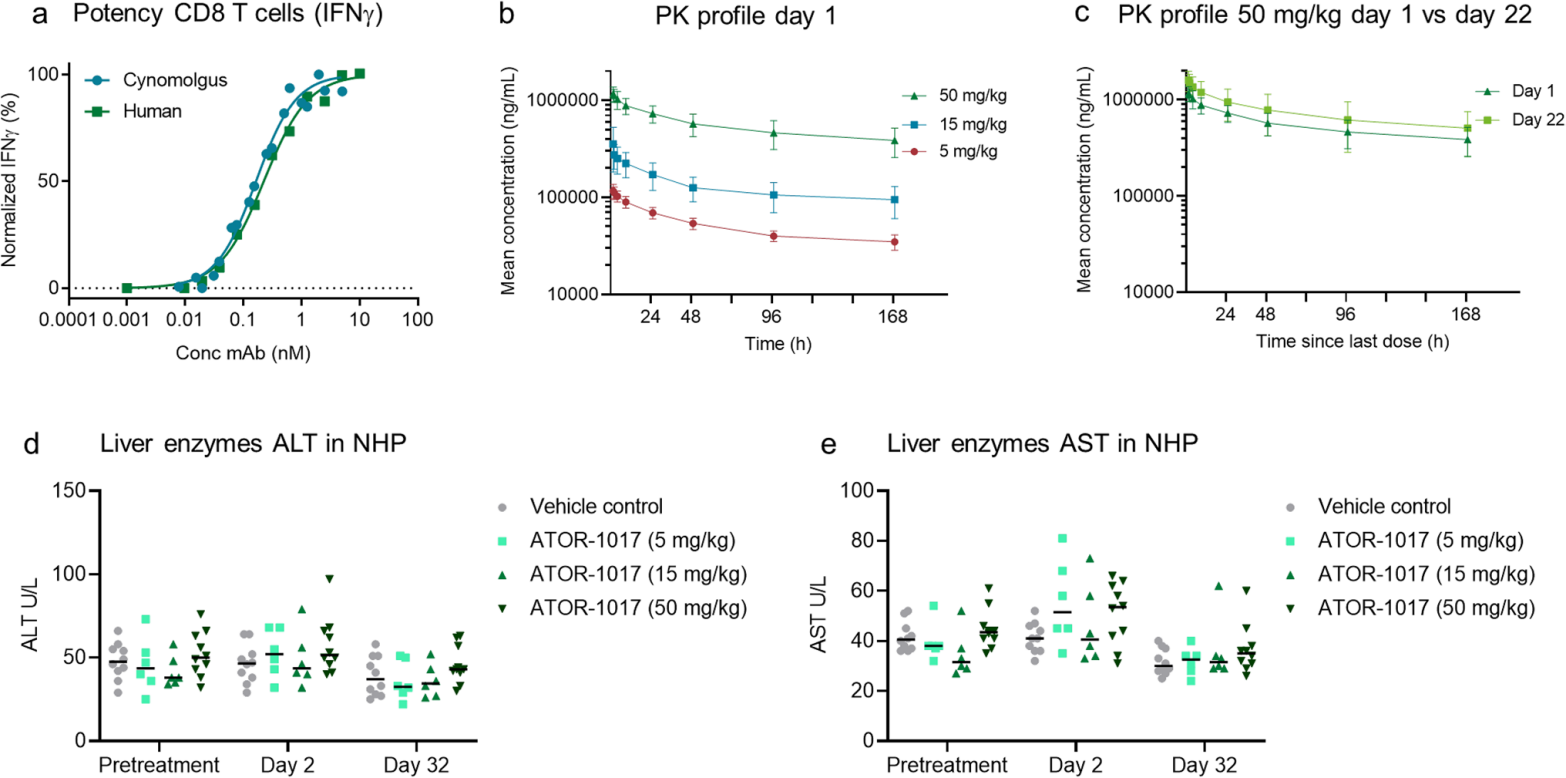
Cyno cross reactivity and Preclinical safety evaluation in NHP **a**, ATOR-1017 induces a comparable dose-dependent activation of both human and cynomolgus CD8 + T cells. FcγRI-CHO cells (0.2 × 10^6^ cells/well) plated overnight were co-cultured with CD8 + T cells activated with CD3-coated beads. ATOR-1017 or isotype control were pre-incubated with CHO cells, prior to the addition of CD3-coated beads and CD8 + T cells (0.1 × 10^6^ cells/well) at a ratio 1:1 for beads and effector cells. After 72 h, cell culture supernatants were harvested and levels of IFNγ were analyzed by ELISA. Mean from five cynomolgus and fifteen human donors are shown. **b-e**, For preclinical safety evaluation, cynomolgus macaques were given 5, once‐weekly, 1‐hour IV doses of ATOR-1017 (5, 15, or 50 mg/kg, *n* = 3 males and 3 females/group) or vehicle control (formulation buffer only, *n* = 3 males and 3 females). On days 1 and 22, pharmacokinetics (PK) was evaluated in all cynomolgus macaques treated with ATOR-1017. Blood samples were obtained at 0 (pre‐dose), 0.5, 1, 3, 8, 24, 48, 96 and 168 h after dosing on Days 1 and 22. ATOR-1017 serum concentration was determined by ELISA. **b**, ATOR-1017 concentration measured in serum after Day 1 in all ATOR-1017 treatment groups (5, 15 and 50 mg/kg of ATOR-1017) **c**, ATOR-1017 concentration measured in serum after Day 1 vs Day 22 in the highest treatment group, 50 mg/kg. Levels of liver enzymes **d**, ALT and **e**, AST before or after treatment of ATOR-1017 in cynomolgus macaques, expressed as absolute values and median (U/L) at pre-treatment, day two and day thirty-two

ATOR-1017 was determined to be fully cross-reactive to cynomolgus macaques and able to augment production of IFNγ by cynomolgus CD8 + T cells in a FcγR-conditional manner, similarly to human CD8 + T cells (Fig. 6a).

In a GLP, 5-week, IV repeat dose toxicology study in cynomolgus macaques, ATOR-1017 was well tolerated ≤ 50 mg/kg/week with no ATOR-1017-related clinical observations, or changes in dose site observations or relevant clinical parameters.

Dose proportional systemic exposure to ATOR-1017 with expected pharmacokinetic profile was observed (Fig. 6b). ATOR-1017 exposure was comparable between Days 1 and 22 at the highest dose of 50 mg/kg (Fig. 6c).

There were no dose-dependent changes in liver enzymes alanine aminotransferase (ALT) and aspartate aminotransferase (AST) following ATOR-1017 treatment on days 2 or 32, with a fold-change to baseline (pre-treatment) below 1.5 for all treatment groups. Values for ALT ranged between 25–76 U/L pre-treatment vs. 22–97 post-treatment and for AST 27–61 U/L pre-treatment vs. 24–81 U/L post-treatment (Fig. 6d-e). All values were considered to be within the normal range for individual variation [38]. Under the conditions of this study, the no observed adverse effect level (NOAEL) was the highest dose evaluated, 50 mg/kg/week.

## Discussion

The distinct and restricted expression of 4-1BB on tumor specific T cells in the tumor environment in combination with the ability of 4-1BB signaling to reactivate T cells with an exhausted phenotype and NK cells makes 4-1BB an attractive target in immuno-oncology [6–10, 39]. However, it is critical to design 4-1BB agonists appropriately to induce activation of tumor specific T cells without inducing systemic T cell activation, which has been associated with liver toxicities [17, 19, 40]. In this study, we describe the development of ATOR-1017, a FcγR-conditional 4-1BB agonistic antibody, designed to outperform the first generation 4-1BB agonists in terms of safety and efficacy. This was achieved by designing an antibody with a 4-1BB binding epitope that depends on FcγR-engagement for its agonistic activity and an IgG4 Fc, which thereby directs the immune activation to the tumor microenvironment where 4-1BB expression is high and there are FcγRs available for Fc engagement and subsequent 4-1BB crosslinking.

The binding epitope of ATOR-1017 was determined by crystallography to be located at domain 2 and 3 overlapping partly with the 4-1BB ligand binding site but distinct from the binding epitope of urelumab as well as utomilumab [19]. The selected binding epitope provides a strong, FcγR-conditional, agonistic activity, in contrast to urelumab that induces agonistic activity also in the absence of FcγR engagement [19]. Further, the superior functional activity of ATOR-1017 compared to utomilumab and other 4-1BB antibodies in IgG4 format, such as ADG106 and CTX471 [19, 29, 30], that bind 4-1BB with similar binding potency indicates that the epitope on 4-1BB is decisive for the agonistic activity of 4-1BB antibodies.

The Fc domain of ATOR-1017 has an IgG4 constant part (S228P stabilized), which allows for effective cross-linking by cells expressing FcγRI and FcγRIIb but very low interaction with cells expressing FcγRIII based on affinity and bioavailability of the FcγR [41]. This is clearly reflected in the ability of ATOR-1017 to induce 4-1BB signaling in the presence of FcγR expressing cells. The choice of Fc confers superior activity compared to utomilumab, which is also cross-linking dependent but has an IgG2 Fc with weak binding to all FcγR, resulting in poor agonistic activity. Further, we demonstrate that ATOR-1017 has a low potential for inducing ADCC of 4-1BB expressing cells (i.e., activated T cells and NK cells) in vitro. We have not detected any signs of ADCC in vivo, and whereas the differences in affinities for mouse and human FcγR (Supplementary Table S1) may be a confounding factor, no depletion of T cells or NK cells was seen in a clinical study with ATOR-1017 [42]. Moreover, in order for ATOR-1017 to bind to FcγRs in the circulation, ATOR-1017 competes with endogenous IgG present at high concentration (approximately 10 mg/L) [43], which lowers the risk for systemic immune activation [44]. Endogenous IgG have been shown to be substantially lower in extravascular compartments [45] and thereby confers less competition and more available FcγRs in tumor tissue vs. the circulation. Therefore FcγR-conditional antibodies, such as ATOR-1017, directs the immune response to the tumor tissue providing an improved therapeutic window compared with FcγR-nonconditional 4-1BB agonists [41]. Considering the wide array of 4-1BB agonistic antibodies that are currently in development [39], selecting the proper Fc is of increasing importance [46] and the role of ADCC remains to be determined [47]. The data presented herein suggests that for a conditional 4-1BB agonist such as ATOR-1017, the IgG4 format provides an opportunity to effectively induce the cross linking required for its agonistic effects without inducing depletion of effector cells.

Treatment of established murine tumors in h4-1BBKI mice with ATOR-1017 resulted in a strong dose-dependent anti-tumor effect as well as an immunological memory to tumor rechallenge. Importantly, while ATOR-1017 treatment induced an increased CD8 + /Treg ratio in the tumor microenvironment, no significant changes in cell numbers were observed in the spleen of any of the parameters analyzed, supporting a tumor directed effect of ATOR-1017. The strong safety profile of ATOR-1017 was further supported by the NHP toxicology study, where ATOR-1017 was well tolerated at doses ≤ 50 mg/kg and clinical data showing that ATOR-1017 was safe and well-tolerated at doses up to 900 mg (flat dose) [42]. Taken together, this data supports our hypothesis that the design of ATOR-1017 confers a tumor directed immune activation with a minimal risk of systemic immune activation.

4-1BB is expressed on a highly relevant population of tumor specific T cells within the tumor microenvironment. However, tumor infiltrating T cells are often in different stages of exhaustion. Activation of exhausted T cells using a tolerable and effective conditional 4-1BB agonistic antibody as described herein has the potential to restore their cytotoxic activity and provide an opportunity to overcome exhaustion as part of anti-PD-1 resistance and potentially increase the response rate in T cell infiltrated cancer indications [10, 48].

Combining anti-PD-1 antibodies with ATOR-1017 is an attractive prospect as these targets induce synergistic anti-tumor activity [2]. In this study, we demonstrate that the ability of anti-PD-1 antibodies to activate T cells with an exhausted phenotype was significantly enhanced when combined with ATOR-1017. Further, this combination induced a stronger anti-tumor activity and increased survival in vivo. Future studies may include combinations with lymphocyte-activation gene 3 (LAG-3) as this combination has been shown to act synergistically to restore function of exhausted T cells and promote tumor regression [10]. In this study, the anti-tumor activity was assessed using one tumor model, MC38, however, mouse specific 4-1BB antibodies have been evaluated in numerous preclinical tumor models and demonstrated synergistic activity with many therapies [39, 49].

Overall, the data presented herein demonstrate that the unique epitope of ATOR-1017 in combination with the choice of an IgG4 Fc has the potential to induce 4-1BB-mediated T cell and NK cell activation in a safe and effective manner. The preclinical data fully supports further clinical development and ATOR-1017 has recently been evaluated in a multicenter, open-label, first-in-human (FIH) phase 1 study (Study No: A-19–1017-C-01, EudraCT No: 2019 001519–21, manuscript in preparation) in patients with advanced and/or refractory solid malignancies to evaluate the safety and tolerability of intravenously (IV) administered ATOR-1017.

### Supplementary Information

Below is the link to the electronic supplementary material.

Supplementary file1 (PPTX 81 kb)

Supplementary file2 (DOCX 34 kb)

## Data Availability

All relevant data is included in the manuscript or supplementary material.
