# Supplementary material for: ATOR-1017 (evunzekibart), an Fc-gamma receptor conditional 4-1BB agonist designed for optimal safety and efficacy, activates exhausted T cells in combination with anti-PD-1
Source: Cancer Immunol Immunother. 2023 Oct 5;72(12):4145–59. doi: 10.1007/s00262-023-03548-7 (PMC10700433; doi:10.1007/s00262-023-03548-7)
Supplement: Supplementary file 1 — Supplementary file1 (PPTX 81 kb) [file 262_2023_3548_MOESM1_ESM.pptx]

## Slide 1
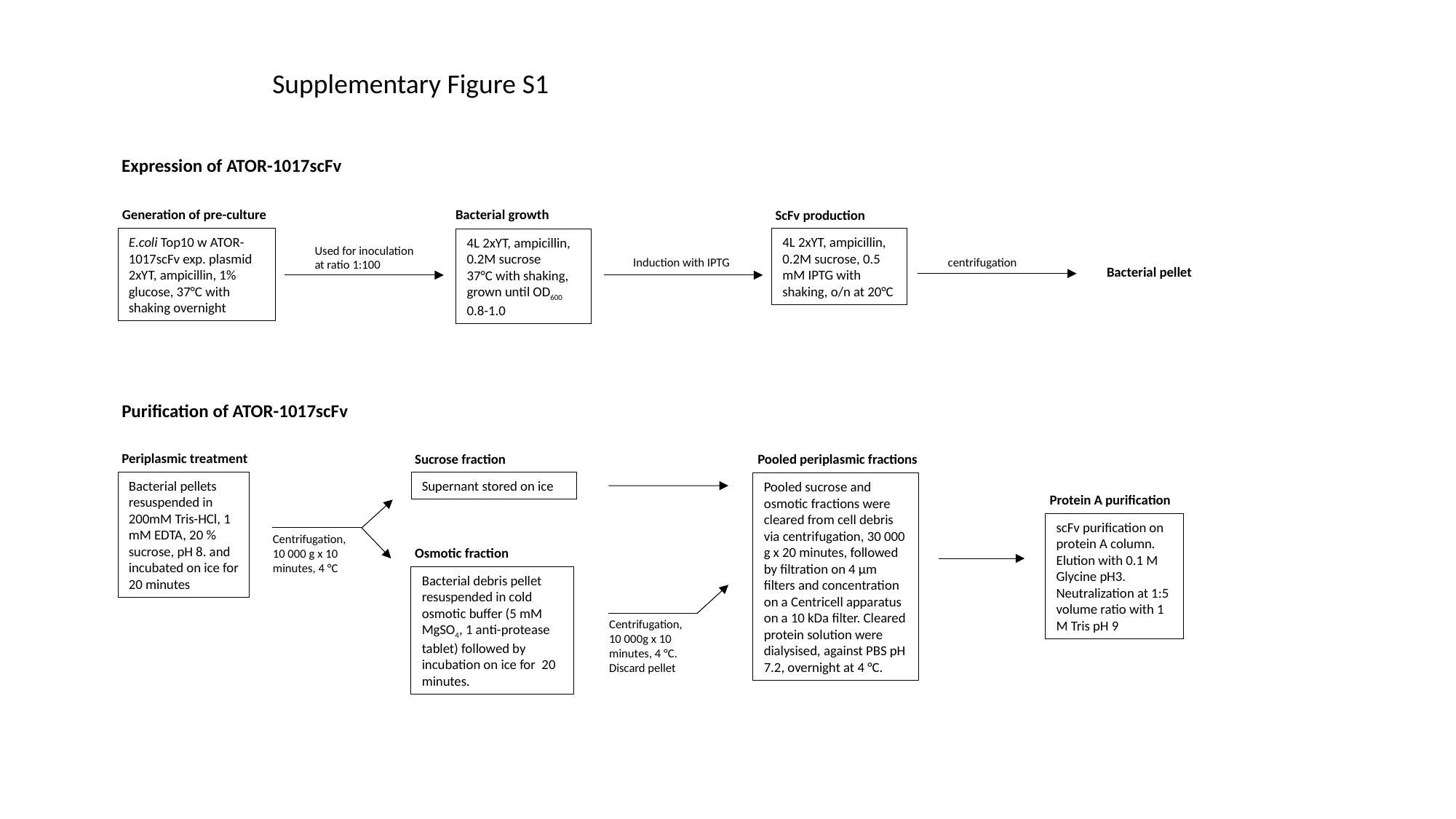

Supplementary Figure S1
Expression of ATOR-1017scFv
Generation of pre-culture
Bacterial growth
ScFv production
E.coli Top10 w ATOR-1017scFv exp. plasmid
2xYT, ampicillin, 1% glucose, 37°C with shaking overnight
4L 2xYT, ampicillin, 0.2M sucrose, 0.5 mM IPTG with shaking, o/n at 20°C
4L 2xYT, ampicillin, 0.2M sucrose
37°C with shaking, grown until OD600 0.8-1.0
Used for inoculation
at ratio 1:100
Induction with IPTG
centrifugation
Bacterial pellet
Purification of ATOR-1017scFv
Periplasmic treatment
Sucrose fraction
Pooled periplasmic fractions
Bacterial pellets resuspended in 200mM Tris-HCl, 1 mM EDTA, 20 % sucrose, pH 8. and incubated on ice for 20 minutes
Supernant stored on ice
Pooled sucrose and osmotic fractions were cleared from cell debris via centrifugation, 30 000 g x 20 minutes, followed by filtration on 4 µm filters and concentration on a Centricell apparatus on a 10 kDa filter. Cleared protein solution were dialysised, against PBS pH 7.2, overnight at 4 °C.
Protein A purification
scFv purification on protein A column. Elution with 0.1 M Glycine pH3. Neutralization at 1:5 volume ratio with 1 M Tris pH 9
Centrifugation, 10 000 g x 10 minutes, 4 °C
Osmotic fraction
Bacterial debris pellet resuspended in cold osmotic buffer (5 mM MgSO4, 1 anti-protease tablet) followed by incubation on ice for 20 minutes.
Centrifugation, 10 000g x 10 minutes, 4 °C. Discard pellet

## Slide 2
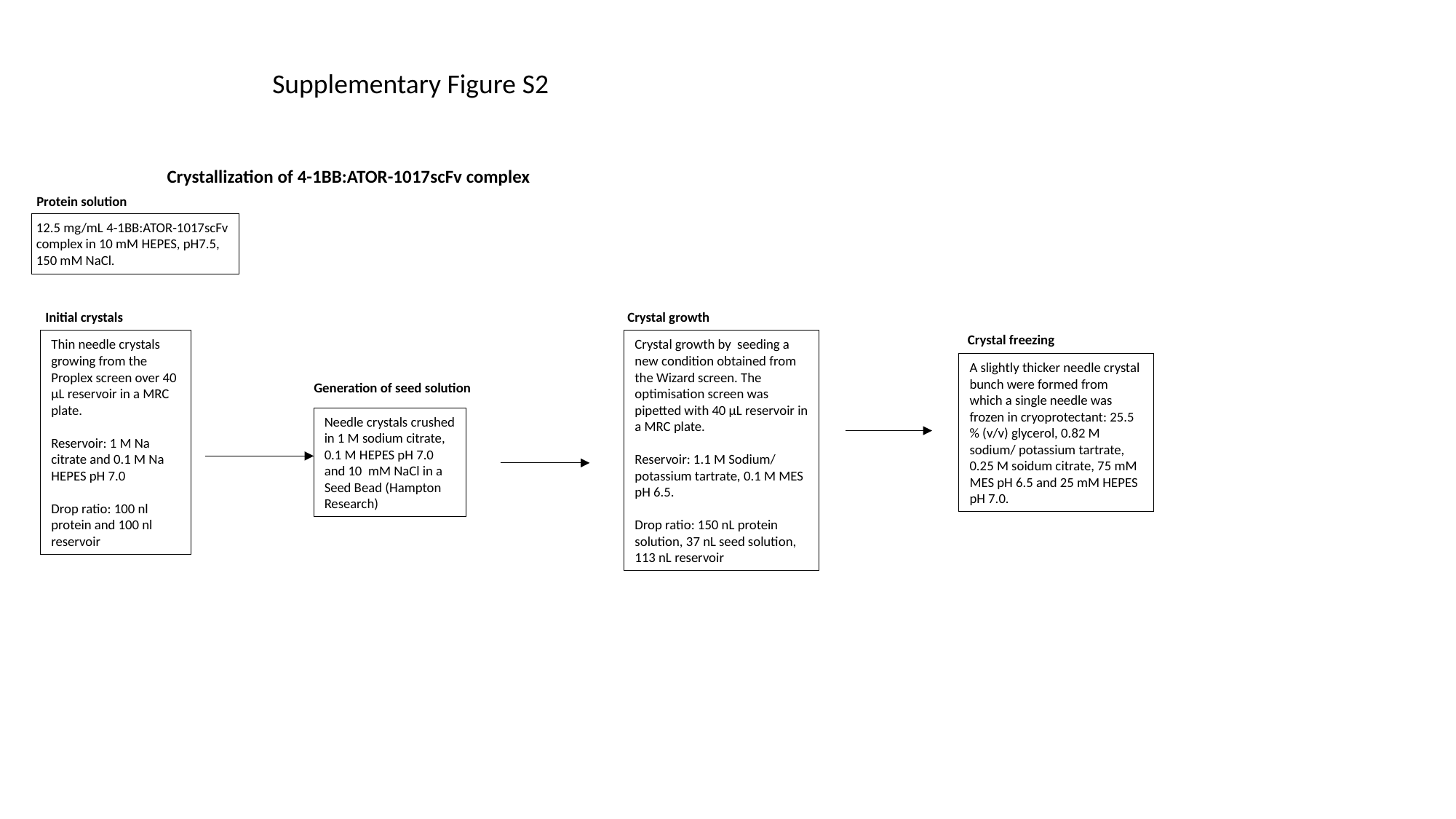

Supplementary Figure S2
Crystallization of 4-1BB:ATOR-1017scFv complex
Protein solution
12.5 mg/mL 4-1BB:ATOR-1017scFv complex in 10 mM HEPES, pH7.5, 150 mM NaCl.
Initial crystals
Crystal growth
Crystal freezing
Thin needle crystals growing from the Proplex screen over 40 µL reservoir in a MRC plate.
Reservoir: 1 M Na citrate and 0.1 M Na HEPES pH 7.0
Drop ratio: 100 nl protein and 100 nl reservoir
Crystal growth by seeding a new condition obtained from the Wizard screen. The optimisation screen was pipetted with 40 µL reservoir in a MRC plate.
Reservoir: 1.1 M Sodium/ potassium tartrate, 0.1 M MES pH 6.5.
Drop ratio: 150 nL protein solution, 37 nL seed solution, 113 nL reservoir
A slightly thicker needle crystal bunch were formed from which a single needle was frozen in cryoprotectant: 25.5 % (v/v) glycerol, 0.82 M sodium/ potassium tartrate, 0.25 M soidum citrate, 75 mM MES pH 6.5 and 25 mM HEPES pH 7.0.
Generation of seed solution
Needle crystals crushed in 1 M sodium citrate, 0.1 M HEPES pH 7.0 and 10 mM NaCl in a Seed Bead (Hampton Research)

## Slide 3
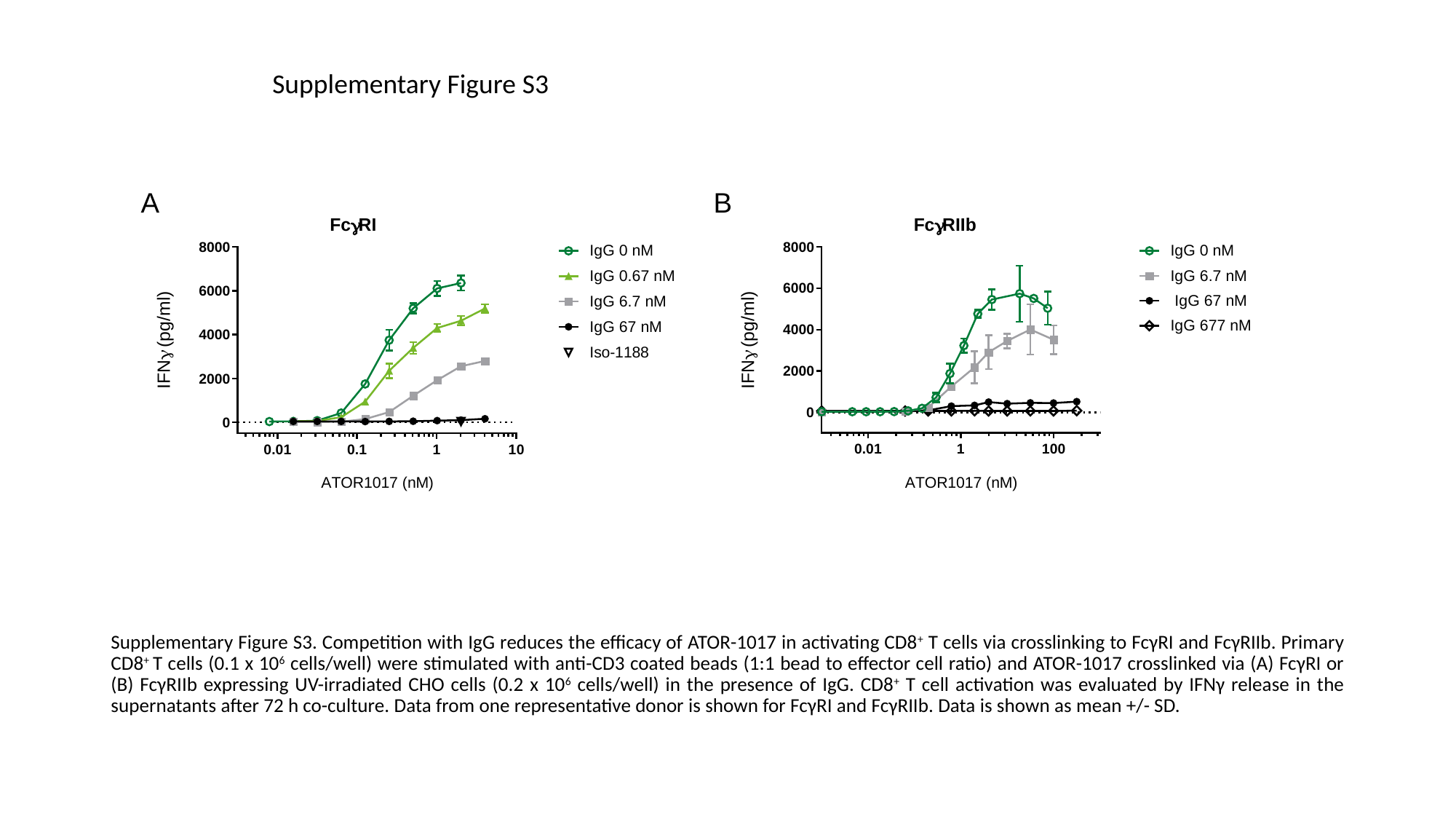

Supplementary Figure S3
Supplementary Figure S3. Competition with IgG reduces the efficacy of ATOR-1017 in activating CD8+ T cells via crosslinking to FcγRI and FcγRIIb. Primary CD8+ T cells (0.1 x 10^6^ cells/well) were stimulated with anti-CD3 coated beads (1:1 bead to effector cell ratio) and ATOR-1017 crosslinked via (A) FcγRI or (B) FcγRIIb expressing UV-irradiated CHO cells (0.2 x 10^6^ cells/well) in the presence of IgG. CD8+ T cell activation was evaluated by IFNγ release in the supernatants after 72 h co-culture. Data from one representative donor is shown for FcγRI and FcγRIIb. Data is shown as mean +/- SD.
