# Supplementary material for: ATOR-1017 (evunzekibart), an Fc-gamma receptor conditional 4-1BB agonist designed for optimal safety and efficacy, activates exhausted T cells in combination with anti-PD-1
Source: Cancer Immunol Immunother. 2023 Oct 5;72(12):4145–59. doi: 10.1007/s00262-023-03548-7 (PMC10700433; doi:10.1007/s00262-023-03548-7)
Supplement: Supplementary file 2 — Supplementary file2 (DOCX 34 kb) [file 262_2023_3548_MOESM2_ESM.docx]

**Supplementary Table S1** Kinetic profiles of ATOR-1017 and a control IgG4 antibody to human and mouse FcγR and FcRn. Kinetic measurements were performed using the Octet RED96 platform (Sartorius), where antibodies were captured on FABG2 Biosensor tips and assayed against recombinant Fc receptors at suitable concentration ranges. Both association and dissociation rates for FcγR as well as association rates for FcRn were studied at physiological pH, while dissociation rates for FcRn were studied at pH6.

|  | **Affinity K_D_ (M) to human FcγR** | | | | | | | | | |  |
| --- | --- | --- | --- | --- | --- | --- | --- | --- | --- | --- | --- |
|  | **hFcγRI** | **hFcγRIIa** | **hFcγRIIb^1^** | | **hFcγRIII 176V^1^** | | | **hFcγRIII 176F** | | **hFcRn** |  |
| **ATOR-1017** | 2 E-09 | 1 E-06 | 8 E-07 | | 1 E-06 | | | <det. limit | | 8 E-07 |  |
| **IgG4 control** | 1 E-09 | 5 E-07 | 6 E-07 | | 6 E-04 | | | <det. limit | | 6 E-07 |  |
|  | **Affinity K_D_ (M) to mouse FcγR** | | | | | | | | | | |
|  | **mFcγRI** | **mFcγRIIb^1^** | | **mFcγRIII** | | **mFcγRIV^1^** | **mFcRn** | |  |  |  |
| **ATOR-1017** | 2 E-07 | 3 E-07 | | 1 E-07 | | 4 E-07 | 2 E-07 | |  |  |  |
| **IgG4 control** | 2 E-07 | 7 E-07 | | 1 E-07 | | 2 E-06 | 2 E-07 | |  |  |  |

^1^Very slow off-rates makes curve fitting difficult.

**Supplementary Table S2**. Statistics on X-ray diffraction data collection and refinement. Frames were measured using a helical scan starting at one part of a single thin rod crystal and ending at another part of the rod in 0.1° oscillation steps at 100K. Numbers in parentheses are statistics for the highest resolution shell.

| **Data collection** | |
| --- | --- |
| Beam line | BioMAX, MAXIV |
| Wavelength (Å) | 0.992 |
| Space group | P22_1_2_1_ |
| **Cell parameters** | |
| A, b , c (Å); α, β, γ (°) | 61.92, 125.21, 128.79; 90, 90, 90 |
| Resolution (Å) | 89.8 – 3.10 (3.31 – 3.10) |
| Unique observations | 18 851 (3345) |
| Multiplicity | 11.7 (12.2) |
| R_merge_ | 0.212 (2.994) |
| R_pim_ | 0.065 (0.887) |
| <I/σ(I)> | 7.5 (1.0) |
| CC(1/2) | 0.997 (0.580) |
| Completeness (%) | 100.0 (100.0) |
| **Refinement** | |
| No. of reflections | 18809 (1274)^1^ |
| R_work_ /R_free_ | 0.205/ 0.271 (0.405 / 0.445)^1^ |
| No. of non-hydrogen atoms | 5473 |
| protein (chains A, B, H, L, C, D) | 5463 |
| solvent | 10 |
| **Geometry** | |
| RMSD values |  |
| bond lengths (Å) | 0.006 |
| bond angles (°) | 1.3 |
| Ramachandran plot quality (%) |  |
| most favoured | 92.9 |
| additionally allowed | 6.7 |
| outliers | 0.4 |
| Rotamer outliers (%) | 2.4 |
| Average B-factor (Å^2^) | 132.4 |

^1^Outer shell of (3.180 - 3.100) Å

**Supplementary Table S3**. Summary of all interacting residues between human 4-1BB and ATOR-1017scFv between 0-4 Å.

| **ATOR-1017** | | **4-1BB** | |
| --- | --- | --- | --- |
| **CDR** | **Residue** | **Residue** | **Domain** |
| HC-CDR1 | Y31^1^ | T89 | 3 |
|  |  | F92^1^ | 3 |
|  | Y33 | G70 | 2 |
| HC-CDR2 | Y59 | K69 | 2 |
| HC-CDR3 | V99 | V71 | 2 |
|  | Y100^1^ | V71^1^ | 2 |
|  | S101^1^ | V71^1^ | 2 |
|  |  | F92 | 3 |
|  |  | C102 | 3 |
|  | S102^1^ | V71^1^ | 2 |
|  | P103^1^ | V71^1^ | 2 |
|  |  | S100^1^ | 3 |
| LC-CDR1 | Q29 | D63 | 2 |
|  |  | I64 | 2 |
|  | S33 | S100 | 3 |
|  | T34^1^ | R66 | 2 |
|  |  | S100^1^ | 3 |
| LC-CDR2 | G52^1^ | M101^1^ | 3 |
|  | S55 | M101 | 3 |
| LC-CDR3 | Y93 | F72 | 2 |
|  |  | S100 | 3 |
|  | Y94^1^ | **F53^1^** | **2** |
|  |  | I64 | 2 |
|  |  | C65 | 2 |
|  |  | R66 | 2 |
|  |  | Q67 | 2 |
|  |  | N83 | 2 |
|  | T95 | Q67 | 2 |
|  | W96^1^ | Q67 | 2 |
|  |  | K69 | 2 |
|  |  | G70^1^ | 2 |
|  |  | F72 | 2 |
|  | V97^1^ | Q67 | 2 |
|  |  | K69^1^ | 2 |

^1^Interaction only present in one of the two 4-1BB:ATOR-1017scFv complexes found in the asymmetric unit
